# Interferons in Autoimmunity: From Loss of Tolerance to Chronic Inflammation

**DOI:** 10.3390/biomedicines13102472

**Published:** 2025-10-11

**Authors:** Grigore Mihaescu, Gratiela Gradisteanu Pircalabioru, Claudiu Natanael Roznovan, Lia-Mara Ditu, Mihaela Maria Comanici, Octavian Savu

**Affiliations:** 1 Department of Botany and Microbiology, Faculty of Biology, University of Bucharest, 030018 Bucharest, Romania; grigore.mihaescu@bio.unibuc.ro (G.M.); claudiu-natanael.roznovan@s.unibuc.ro (C.N.R.); lia-mara.ditu@bio.unibuc.ro (L.-M.D.); 2 Research Institute of University of Bucharest, 050663 Bucharest, Romania; 3 eBio-Hub Centre of Excellence in Bioengineering, National University of Science and Technology Politehnica Bucharest, 060042 Bucharest, Romania; 4 Department of Preclinical Sciences, Faculty of Medicine, Titu Maiorescu University, 040441 Bucharest, Romania; mihaela-maria.pischis@s.unibuc.ro; 5 “N.C. Paulescu” National Institute of Diabetes, Nutrition and Metabolic Diseases, 020042 Bucharest, Romania; octavian.savu@umfcd.ro; 6 Department of Doctoral School, “Carol Davila” University of Medicine and Pharmacy, 5th District, 050474 Bucharest, Romania

**Keywords:** interferons, cytokines, autoimmune diseases, type I IFN, type II IFN, type III IFN, interferon signature, inflammation, immune tolerance, immunotherapy

## Abstract

Interferons (IFNs) are key cytokines at the intersection of innate and adaptive immunity. While their antiviral and antitumor roles are well recognized, emerging evidence implicates IFNs—particularly types I, II, and III—in the initiation and progression of autoimmune diseases (ADs). This review synthesizes current data on IFN biology, their immunoregulatory and pathogenic mechanisms, and their contributions to distinct AD phenotypes. We conducted a comprehensive review of peer-reviewed literature on IFNs and autoimmune diseases, focusing on publications indexed in PubMed and Scopus. Studies on molecular pathways, immune cell interactions, disease-specific IFN signatures, and clinical correlations were included. Data were extracted and thematically organized by IFN type, signaling pathway, and disease context, with emphasis on rheumatic and systemic autoimmune disorders. Across systemic lupus erythematosus, rheumatoid arthritis, Sjögren’s syndrome, systemic sclerosis, idiopathic inflammatory myopathies, multiple sclerosis, type 1 diabetes, psoriasis, and inflammatory bowel diseases, IFNs were consistently associated with aberrant activation of pattern recognition receptors, sustained expression of interferon-stimulated genes (ISGs), and dysregulated T cell and B cell responses. Type I IFNs often preceded clinical onset, suggesting a triggering role, whereas type II and III IFNs modulated disease course and severity. Notably, IFNs exhibited dual immunostimulatory and immunosuppressive effects, contingent on tissue context, cytokine milieu, and disease stage. IFNs are central mediators in autoimmune pathogenesis, functioning as both initiators and amplifiers of chronic inflammation. Deciphering the context-dependent effects of IFN signaling may inform targeted therapeutic strategies and advance precision immunomodulation in autoimmune diseases.

## 1. Introduction

The functional balance of the immune system (IS), reflected in its tolerance towards self and reactivity towards non-self, is mainly maintained by immune cells such as B and T lymphocytes, dendritic cells (DCs), and macrophages [1]. Loss of tolerance towards self is the basis for autoimmune (AI) conflicts, potentially progressing towards autoimmune diseases (ADs). The pathogenesis of an AD can be initiated by immune dysregulation stemming from genetic predisposition, precipitating events such as infectious processes, or a state of immunosuppression [2].

Autoimmune diseases (ADs) arise from the convergence of genetic susceptibility, environmental exposures, and dysregulated immune circuits that collectively breach self-tolerance. Susceptibility is strongly shaped by HLA variation, with class II alleles (e.g., HLA-DRB1 shared epitope in rheumatoid arthritis; HLA-DRB1/DQA1/DQB1 in systemic lupus erythematosus and Sjögren’s syndrome) dictating peptide presentation and T cell help to autoreactive B cells [3]. Beyond HLA, numerous non-HLA risk loci modulate innate and adaptive signaling thresholds: PTPN22 and CTLA4 alter lymphocyte activation and checkpoints; STAT4, IRF5, TYK2, and TNFAIP3 (A20) tune cytokine and NF-κB pathways; and BLK and BANK1 affect B cell receptor signaling and plasmablast differentiation. Genes governing nucleic acid metabolism and sensing provide a mechanistic bridge to interferon biology: rare and common variants in TLR7, IFIH1 (MDA5), TREX1, ADAR1, DNASE1L3, RNASEH2, and TMEM173 (STING1) potentiate accumulation or recognition of self-derived nucleic acids, lowering the threshold for type I interferon (IFN) production and imprinting an “IFN-high” endotype in subsets of SLE, dermatomyositis, and related conditions [4,5,6,7]. These germline risks interact with epigenetic remodeling—global and locus-specific DNA hypomethylation, histone modifications, and microRNA networks—that reprogram myeloid and lymphoid cells toward heightened interferon responsiveness [8]. Environmental drivers further modulate disease expression: viral exposures (notably EBV), microbiome dysbiosis, cigarette smoke, silica and occupational particulates, ultraviolet light, and hormonal milieu (including X chromosome dosage effects) can precipitate or amplify flares [9,10,11]. Mechanistically, defective clearance of apoptotic and neutrophil extracellular trap (NET) debris, impaired complement opsonization (C1q/C2/C4 deficiency), and persistent activation of endosomal and cytosolic nucleic acid sensors (TLR7/9; cGAS–STING; RIG-I/MDA5) converge on plasmacytoid dendritic cells and tissue stroma to sustain IFN programs [12]. The downstream consequences—ISG upregulation, enhanced antigen presentation, and co-stimulatory licensing—promote autoreactive B cell maturation and pathogenic T cell help, anchoring interferon pathways as central organizers of disease heterogeneity across ADs.

Genetic predisposition, reflected in the increased incidence of certain ADs associated with homozygous expression of certain MHC class II alleles, appears to play a determining role in the onset of autoimmune conflicts [5]. The latent autoimmune genetic potential, in the context of an undefined number of predisposing alleles, can become functional upon activation of self-reactive lymphocytes in response to antigens of infectious agents that cross-react with self-antigens [13,14,15]. The risk of generating immune effectors of the autoimmune conflict is facilitated by chronic inflammatory processes, during which proinflammatory cytokines IL-1, IL-6, TNF, and IL-18 induce a state of immunosuppression [16].

In the pathological process of ADs, autoimmune reactivity leads to the lysis of target tissue cells, resulting in the release of a broad spectrum of antigens that generate persistent immune complexes, including chromatin and RNA, which stimulate pattern recognition receptors (PRRs) and trigger the synthesis of interferons (IFNs) [17].

The aim of this study is to analyze a group of ADs characterized by decreased Treg activity and markedly increased serum levels of interferons (IFNs), which, through the large number of genes they stimulate (ISGs—interferon-stimulated genes), lead to pathological manifestations of varying severity.

## 2. Lymphocytes Regulating Immune Function: Loss of Tolerance

Both T and B lymphocyte populations carry out effector and regulatory functions. The regulation of the intensity and duration of the immune response is the result of a quantitative balance of a multitude of pro- and anti-inflammatory ILs, primarily released by T lymphocytes, although B lymphocytes also play an important regulatory role [18].

T lymphocytes represent 70–80% of the total circulating lymphocytes. Mature T lymphocytes express either the CD4 or CD8 marker and comprise distinct, functionally heterogeneous subpopulations: they fulfill both effector functions of the cell-mediated immune response and regulatory roles through autocrine- or paracrine-acting lymphokines [19].

In humans, CD4^+^ T lymphocytes account for 55–65% of the total number of T lymphocytes. They synthesize a wide variety of ILs through which they control the functioning of the IS.

Based on the set of lymphokines they synthesize, CD4^+^ T lymphocytes are classified into at least four subsets: Th1, Th2, Treg, and Th17, all of which derive from precursor Th0 cells. The most numerous subpopulation of CD4^+^ T cells are the helper T lymphocytes (Th cells):

1. Th1 lymphocytes play a role in establishing the cell-mediated immune response (CMIR). Their response to intracellular infectious agents involves the secretion of proinflammatory interleukins that stimulate CMIR: IL-2, IFN-γ, and lymphotoxin (LTα = TNF-β). IFN-γ and LTα activate the phagocytic function of macrophages and the production of nitric oxide (NO). IL-2 stimulates the proliferation of CD8^+^ T lymphocytes, which are effectors of CMIR, and also the synthesis of small amounts of IL-4 [20]

2. Th2 lymphocytes (Th-b) differentiate in response to IL-4. Under the influence of IL-4, Th2 cells secrete type 2 cytokines: IL-4, IL-5, IL-10, and IL-13. They do not secrete lymphotoxins but do synthesize small amounts of IL-2 [21]. IL-4 and IL-5 stimulate the differentiation of B lymphocytes into plasma cells and the synthesis of antibodies against infectious agents. The ILs secreted by Th1 and Th2 lymphocytes regulate the dynamics of the immune response. Th1 cells release proinflammatory ILs and stimulate CMIR, while Th2 cells produce anti-inflammatory cytokines, inhibit CMIR, stimulate humoral immune response (HIR), and mediate immune responses in allergies.

3. Treg lymphocytes (regulatory T cells) express the markers CD3+, CD4+, CD25+, and Foxp3+ (Forkhead box P3). Foxp3+ is a transcriptional regulatory factor essential for the differentiation of Treg cells and is their most reliable marker. The differentiation of Foxp3+ cells is IL-2-dependent: IL-2 increases Foxp3+ expression levels and stimulates the growth, division, and activity of Treg cells. IL-4 can partially compensate for IL-2 deficiency [22]. Another factor in Foxp3 expression is type I IFN, whose synthesis by DCs is stimulated by the intestinal microbiota. The FOXP3 gene is regulated by type I IFNs via ISGs [23].

Treg cells are suppressors of the IR, their function depending on the high expression of the Foxp3+ marker. Treg cells reduce the intensity of both humoral and cellular immune responses after antigen (Ag) clearance, maintaining immune reactivity within physiological limits. They suppress the activity of Th1 and Th2 lymphocytes primarily through the synthesis of inhibitory ILs that regulate immune checkpoints, the production of anti-inflammatory cytokines (IL-10, IL-35, TGF-β), and through enzymes that generate adenosine, whose catabolites are toxic [24].

The immunosuppressive function of Treg lymphocytes is essential for maintaining tolerance to self-antigens and is regulated by the balance of pro- and anti-inflammatory cytokines that sustain Foxp3 expression. Immunosuppression beyond physiological limits extends tolerance to non-self-antigens, especially those with a certain degree of similarity to self-molecules. Excessive activation of Treg cells can lead to generalized immunosuppression and a reduction in the intensity of immune responses, including those against tumor antigens.

Many ADs are associated with downregulated Treg function caused by mutations in the FoxP3 gene, along with increased Th1, Th2, and Th17 activity, which play a role in the pathogenesis of many ADs. This reflects a loss of Treg control [25] and supports the concept of ADs as chronic, proinflammatory “low Treg” conditions that favor the initiation of autoimmune conflict. Abnormal Th2 lymphocyte expression is associated with antibody-mediated ADs and increased synthesis of cytokines and chemokines [26]. IPEX syndrome (immune dysregulation, polyendocrinopathy, enteropathy, X-linked syndrome) represents a group of rare recessive disorders caused by FoxP3 gene mutations [27].

4. Th17 lymphocytes are cells of mucosal tissues, scarce in the circulating blood of healthy or neoplastic organism. They are defined by the synthesis of IL-17A, IL-17 F, IL-21, and IL-22, which Th1 or Th2 cells do not produce.

Along with decreased Treg function, Th17 frequency and function upregulates in MS, psoriasis, RA, IBD, and SLE.

Higher Th17 ratio and function is associated with the development and progression of AI processes and inflammatory bowel pathology (ulcerative colitis, Crohn’s disease), characterized by T cell infiltration in mucosal tissues, as well as multiple sclerosis (MS), psoriasis, RA, and SLE [28,29]. Unlike terminally differentiated Th1 and Th2, Th17 lymphocytes are versatile, they can (1) differentiate into proinflammatory Th1 or IR suppressor Treg Foxp3+ lymphocytes, depending on the quantitative ratio of cytokines and chemokines at the inflammation site as well as ratio of other infiltrating leukocytes; and (2) differentiate into IFNγ-synthesizing cells (Th1), by repressing the gene site for IL-17 synthesis and increasing the expression of the site encoding IFNγ [30].

### Differentiation of CD4^+^ T Lymphocyte Subsets

**Figure 1:**
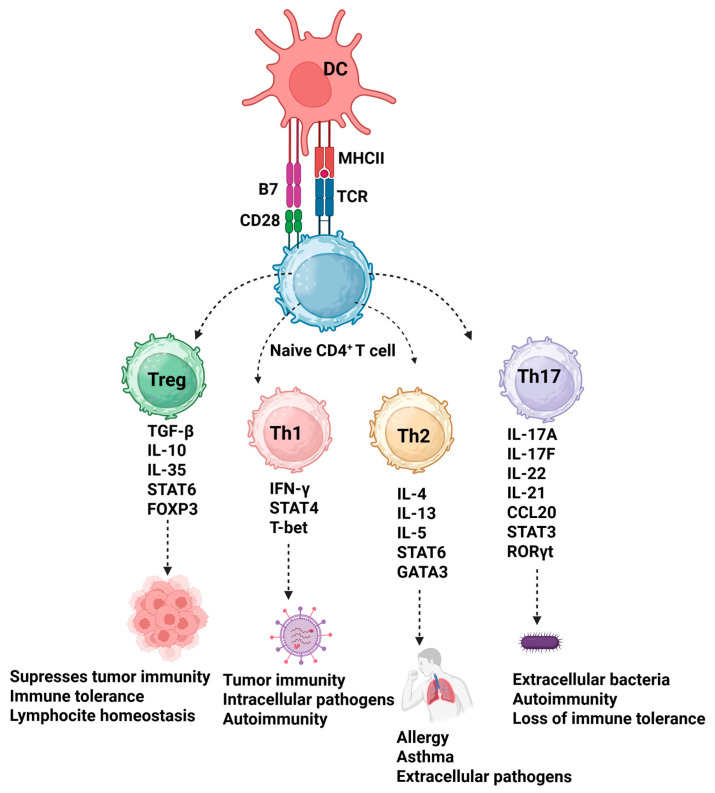
Differentiation of naïve CD4^+^ T cells into effector and regulatory subsets. Naïve CD4^+^ T cells activated by antigen-presenting dendritic cells (DCs) through MHCII–TCR interaction, CD28 co-stimulation, and B7 signaling can differentiate into distinct lineages depending on the cytokine milieu and transcription factor activation. Regulatory T cells (Treg) are driven by TGF-β, IL-10, and IL-35, under STAT6 and FOXP3 control, and function to suppress tumor immunity, maintain immune tolerance, and regulate lymphocyte homeostasis. Th1 cells develop under IFN-γ and STAT4 signaling, mediated by T-bet, and are involved in tumor immunity, defense against intracellular pathogens, and autoimmunity. Th2 cells are induced by IL-4, IL-13, and IL-5, under STAT6 and GATA3 control, and mediate allergic responses, asthma, and defense against extracellular pathogens. Th17 cells differentiate in the presence of IL-17A, IL-17F, IL-22, IL-21, and CCL20, regulated by STAT3 and RORγt, and are associated with extracellular bacterial defense, autoimmunity, and loss of immune tolerance.

Naïve (uncommitted) CD4^+^ T lymphocytes differentiate into distinct effector subsets under the influence of cytokines such as IL-6, IL-21, and TGF-β [31]. Th1 cells produce IFN-γ, IL-2, lymphotoxin-α (LT-α, also known as TNF-β), and in some cases, TNF-α. They promote cell-mediated immunity (CMI), particularly effective against intracellular pathogens (Figure 1). Th2 cells, which do not synthesize IFNs, secrete IL-4, IL-5, IL-10, and IL-13. These cytokines are associated with humoral-mediated immunity (HMI) and are involved in responses to extracellular parasites and allergic inflammation. Th17 cells are characterized by the expression of the transcription factors RORγt and STAT3. Their lineage commitment is stabilized by IL-1β and IL-23. Th17 cells produce IL-17A, IL-17F, IL-21, and IL-22, playing critical roles in mucosal defense, inflammation, and the pathogenesis of autoimmune diseases. The differentiation of Th1, Th2, and regulatory T cells (Tregs) is governed by IL-12, IL-4, and TGF-β, respectively, along with the transcription factors T-bet (Th1), GATA3 (Th2), and FoxP3 (Treg). Th2, Th17, and Treg subsets contribute to the regulation of immune responses against tumor-associated antigens, self-antigens, and environmental antigens [32].

The population of B lymphocytes is functionally heterogeneous: after activation following Ag stimulation; they differentiate and become primarily Ac-producing plasmocytes. Others produce IR modulatory cytokines, a function mediated by IL. Regulatory B cells produce proinflammatory IL: IL-1β, IL-6, TNF, IFN γ, GM-CSF, T cell stimulators, and anti-inflammatory IL-10 [33]. Functional heterogeneity is explained by the specificity of the activating signal. B lymphocytes activated by the BCR-CD40 complex release proinflammatory ILs, whereas those stimulated only by the CD40 coreceptor synthesize the anti-inflammatory IL-10 [34,35].

Neutrophils in ADs are mainly proinflammatory, examples in this regard being RA and SLE. In RA, neutrophils infiltrating the joints carry out cytotoxic roles through secreted lysosomal enzymes, at the same time recruiting other neutrophils to the inflammation sites, while circulating neutrophils are responsible for ROS production. NETs, released by neutrophils, contain citrullinated histones and may be associated with TNF secretion [36].

Macrophages are heterogeneous in both origin and function. The majority of tissue macrophages are not derived from circulating monocytes, but are of embryonic origin [37]. Macrophages, similar to neutrophils, have a proinflammatory phenotype (M1) in SLE, RA, D1, etc., but in fibrotic ADs (SSc, MS, IBD) a relatively large proportion have both M1 and M2 markers [38,39,40]; this is explained by epigenetic changes reflected in transcription [41]. In fact, macrophages form a functional continuum of reversible M1-M2 activation states, depending on the AI progression, infectious or neoplastic pathological process [42].

Neutrophils and activated monocytes release ROS. Oxidative stress decreases Treg numbers, diminishes immunosuppression and intensifies the progression of some ADs (SLE). Central tolerance, established in the thymus via deletion of autoreactive T cell clones, is essential but not absolute. As many as 40% of naïve T cells leaving the thymus retain low-affinity self-reactivity and can promote autoimmunity, particularly in immunosuppressed settings. As a result, immune tolerance is also controlled by peripheral mechanisms [43].

The steady-state of immune function and the inflammatory response are regulated by a large number of cytokines, categorized into six families according to the biological effects they mediate: ILs, IFNs, TNFs, chemokines, TGFs (transforming growth factors), and hematopoietic growth factors.

A key factor in controlling peripheral tolerance is TGF-β, a pleiotropic cytokine, produced mainly by T cells. The defining function of TGF-β is maintaining the suppression of autoreactive T cells to a physiological level, but the effects are dependent on the differentiation stage of the T cells and the conditions of stimulation [44]. TGF-β controls the immune tolerance state directly by inducing FoxP3+ expression in peripheral Treg cells and indirectly by stimulating Th17 conversion to immunosuppressive TregFoxP3+. TGF-β deficiency induces AI conflicts.

Genetic studies confirm the association of ADs with certain MHC II allelic combinations. Some alleles increase the predisposition towards ADs (positive association), while others reduce the risk of ADs (negative association). In ADs, MHC II molecules are overexpressed and elicit proinflammatory responses [45].

Genetic predisposition for some ADs, generated by a particular gene combination is potentiated by immune function imbalance. The most common haplotypes associated with ADs are DR3-DQ2 and DR4-DQ8, while the generally protective allele is HLA-DRB1*0701 [46].

Regardless of the genetic determinism, some ADs are pathological conditions characterized by low Treg levels and intense inflammatory responses, both of which activate Th1 and Th17 lymphocytes, implicated in the pathogenesis of many ADs [47,48]. The downregulation of immunosuppressive function leads to increased tolerance, another condition favoring the expression of predisposing alleles with pathological potential [49].

## 3. Interferons

**Figure 2:**
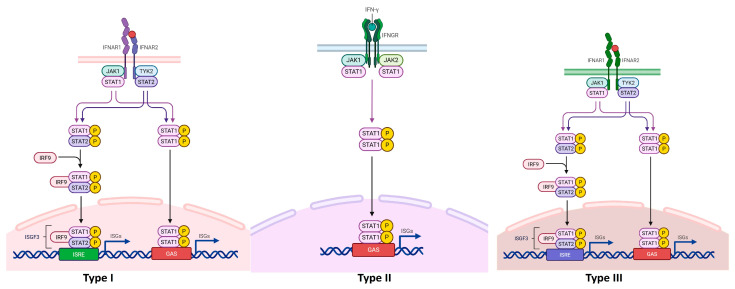
Canonical signaling by type I, II, and III interferons. **Left** (Type I): Type I IFNs (IFN-α/β/κ/ε/ω) bind the heterodimeric IFNAR1/IFNAR2 receptor and activate JAK1 and TYK2, leading to phosphorylation of STAT1 and STAT2. Phospho-STAT1/STAT2 complexes associate with IRF9 to form ISGF3, which translocates to the nucleus and binds ISRE elements to induce interferon-stimulated genes (ISGs). Type I IFNs can also generate STAT1 homodimers that bind GAS elements. **Middle** (Type II): IFN-γ engages IFNGR1/IFNGR2 and activates JAK1/JAK2, resulting in STAT1 phosphorylation and formation of STAT1 homodimers that bind GAS to drive gene expression. **Right** (Type III): IFN-λ1/2/3/4 signal through IFNLR1/IL10RB with JAK1/TYK2, predominantly in epithelial/barrier tissues. Downstream, ISGF3 binding to ISRE and STAT1 homodimers binding to GAS drive context-dependent ISG programs. P denotes phosphorylation. Abbreviations: IFN, interferon; IFNAR, IFN-α/β receptor; IFNGR, IFN-γ receptor; IFNLR, IFN-λ receptor; JAK, Janus kinase; TYK2, tyrosine kinase 2; STAT, signal transducer and activator of transcription; IRF9, interferon regulatory factor 9; ISGF3, interferon-stimulated gene factor 3; ISRE, interferon-stimulated response element; GAS, gamma-activated sequence; and ISG, interferon-stimulated gene.

Interferons form a large family of cytokines, classified based on serological and phylogenetic criteria into four types: I, II, III, and IV (Figure 2). In placental mammals, type I IFN group 9 variants include the following: (i) IFN-α (13 subtypes), (ii) IFN-β, (iii) IFN-ε, (iv) IFN-ξ (limitin), (v) IFN-κ, (vi) IFN-ω (in cattle and humans), (vii) IFN-δ (in pigs), (viii) IFN-ϛ, and (ix) IFN-τ (trophoblastic, in ruminants) [50,51,52]. IFN-u described in Danio rerio (zebrafish) is considered, on phylogenetic grounds a type IV IFN, because its receptors (IFNuR1/IL10R2) are common to class II cytokine receptors, which include IFN types I, II, and III and their receptors.

The genes encoding IFNs have evolved by duplication [53]. Each type of IFN is synthesized by particular categories of cells in response to specific stimuli.

The human body synthesizes 17 subtypes of IFN-I: 13 IFN-α subtypes and one of each β, ω, κ, and ε subtypes.

The synthesis of the different subtypes depends on the cell type and the cytokines present in the cellular microenvironment. Thus, pDCs produce IFN-α in response to PAMP or DAMP signals, and keratinocytes constitutively synthesize IFN-κ, a synthesis amplified by psoriasis pathology and tissue regeneration [54]. IFN-ε is synthesized by the endometrial epithelium and is regulated by estrogen and progesterone in the menstrual cycle [55].

IFNs are the bridge between trigger factors and autoimmune reactivity. IFN synthesis is, in fact, a response to stimuli recognized by innate immunity sensors TLR, RIG-I and MDA-5, NLR, cGAS-STING (cyclic GMP-AMP-synthetase-stimulator of IFN Genes) and ALR (AIM 2 like-receptor). The most important sensor for the IFN activating pathway is TLR-7 [56].

Type I and type III IFN synthesis is triggered by signals released consequently to PRR sensors recognizing exogenous DNA and RNA in the cytosol or endosome, or by endogenous nucleic acids.

IFN-α is synthesized by most nucleated cells, including circulating leukocytes, but pDCs are the major producers [57]. IFN-b is synthesized by virus-infected or dcRNA-stimulated fibroblasts and other activated cells [58].

Type III IFNs (λ1–λ4) are primarily synthesized by epithelial cells of the respiratory tract, digestive tract, endothelial cells of the brain and kidney, and cells of hematopoietic origin [51]. The four variants, λ1/IL-29; λ2/IL-28A; λ3/IL-23B; and λ4 are encoded by closely linked genes on chromosome 19. λ4 is a functional pseudogene in a fraction of the population [59]. All four λ variants bind to the common double-stranded receptor λR1/IL-10R2.

Structurally and by specific receptors, IFN-λ differs greatly from IFN-I and -II, but has some similarities with IFN-I: their synthesis is induced by viral infection, and they activate the same JAK-STAT signaling pathway and IRFs, and induce transcription of the same set of ISGs [60,61].

IFN-λ has important functions in innate and adaptive IR and may be involved in pathogenetic AI processes.

Although it was initially considered that IFN-λ has no proinflammatory effect and that its function was limited to a protective effect of epithelial surfaces from viral, bacterial and fungal respiratory infections [62,63], subsequent results reported that IFN-λ, like IFN-I, stimulates CMI and the transcription of proinflammatory cytokines [64].

IFN type II (IFN-γ) synthesis is not induced by PRR sensors, but by IL-12 and IL-18. Activated macrophages release IL-12, stimulating immune cells like NK, NKT, TCD4^+^, TCD8^+^αβ, Tγδ TCD4^+^, activated B cells and neutrophils. It is considered an IR-stimulatory and proinflammatory IFN [65,66,67]. IFN-γ binds to a universal heterodimeric receptor, IFNGR (subunits IFNGR1 and IFNGR2), expressed on most cell types. Of the four JAK kinases -1, -2, -3 and TYK2, IFNGR1 and IFNGR2 associate with JAK1 and JAK2, respectively. All four kinases have a C-terminal catalytic domain and an enzymatically inactive domain—a pseudokinase. Phosphorylation of tyrosine residues is the essential step for activation of the essential JAK/STAT pathway [68]. ISGs trigger signal propagation. The three types of IFNs bind to distinct receptors, but converge on the major common JAK-STAT (Janus kinase/Signal transducer and activator of transcription) signaling pathway and activate ISGs and IRFs, thereby stimulating responses which explains why different IFNs stimulate a common set of genes [69].

The specific IFN type I receptors—IFNAR1 and IFNAR2—dimerize and induce autophosphorylation of JAK1 and TYK2. The two autophosphorylated kinases phosphorylate tyrosine residues of STAT effector proteins. There are seven STAT proteins (-1, -2, -3, -4, -5A, -5B, -6) [70].

JAK1 and TYK2 phosphorylate STAT1 and STAT2, and form STAT1/STAT1 homodimers or STAT1/STAT2 heterodimers. Dimers, upon activation, recruit IRF9 and form the STAT1-STAT2/IRF9 heterotrimer complex, known as ISGF3 (IFN-stimulated gene factor 3) [71]. The ISGF3 complex, upon translocation into the nucleus, activates the ISRE (IFN-stimulated response element) of the ISG promoter and initiates transcription.

Phosphorylation occurs not only at tyrosine residues but also at serine residues, while STAT activation and transfer to the nucleus without phosphorylation has also been observed [72]. There are also other signaling pathways, which induce various IFNγ receptor actions [66].

The IGNGR1 and IFNGR2 receptors of IFN-γ phosphorylate JAK kinases, which in turn phosphorylate different dimeric combinations of the seven STAT proteins: STAT1/STAT1, STAT2/STAT2 homodimers or STAT1/STAT2, STAT1/STAT3/4/5/6 heterodimers [73,74,75]. Dimers associate with IRFs (IFN Regulatory Factors), after which the trimeric complex is translocated into the nucleus and interacts with GAS (Gamma IFN Activation Sequence) elements. This produces a marked polymorphism of IRFs that diversifies the number of ISGs [76]. Out of about 1600 transcription factors, the IRFs activated by IFNs make up an unknown proportion [76].

IFN-γ has pleiotropic action; it modulates the expression of about 2339 genes, many of which are also controlled by IFN-I. The effects of IFN-γ on target genes are heterogeneous, stimulatory or inhibitory, depending on tissue specificity and interaction with other cytokines and metabolic rate, which diversifies its effects on the development of Ads [77].

IFNs are modulators of IR, have a protective antiviral role, but may accentuate pathogenicity of ADs [78].

IFNs have major roles, both direct and indirect, in regulating gene expression. Some ISGs transcribe non-coding RNAs, such as miRNAs and lncRNAs, which regulate gene expression [79,80].

Production of IFN is both subset- and tissue-specific (Table 1). Plasmacytoid dendritic cells (pDCs) are the dominant acute source of type I IFNs (particularly IFN-α) following endosomal TLR7/9 ligation, and they orchestrate the systemic “IFN-high” endotype seen in lupus and related conditions [81,82]. Conventional dendritic cells (cDC1/cDC2) and inflammatory monocytes/macrophages contribute type I IFN under cytosolic sensing of nucleic acids (RIG-I/MDA5–MAVS; cGAS–STING), while keratinocytes and other stromal cells provide tissue-resident IFN tone (e.g., IFN-κ in skin, IFN-β in fibroblasts and epithelial cells) [83]. Epithelial cells at barrier sites are the principal source and target of type III IFNs (IFN-λ), creating an epithelium-restricted antiviral program. Activated T and NK cells produce type II IFN (IFN-γ), which licenses macrophage effector functions and shapes antigen presentation. The magnitude and kinetics of IFN release differ across subsets—pDCs deliver rapid, high-amplitude pulses, and stromal and epithelial compartments sustain lower but spatially focused signaling—so disease phenotypes often reflect the dominant cellular niche engaged in a given organ [84].

Expanding disease coverage beyond SLE, RA, and Sjögren’s syndrome clarifies how interferon biology diversifies across autoimmune settings. In systemic lupus erythematosus and cutaneous lupus, pDC-driven type I IFN and keratinocyte-derived IFN-κ form a feed-forward loop that elevates ISG signatures in blood and skin; this axis is therapeutically tractable, as evidenced by clinical benefit with IFNAR blockade and by pharmacodynamic suppression of ISGs [85]. Primary Sjögren’s syndrome features glandular and peripheral IFN-I signatures arising from epithelial and pDC compartments, closely linked to CXCL10/CXCL13 chemokines and ectopic germinal center features. In rheumatoid arthritis, IFN-γ and, in subsets, IFN-I contribute to synovial myeloid activation and GAS/ISRE-driven programs; downstream JAK inhibition partially mitigates these effects even when IFN is not the sole driver [86]. Systemic sclerosis maintains a consistent IFN-I signature across blood and skin, likely reflecting pDC and fibroblast circuits, with chemokines such as CXCL10 correlating with severity [87]. Dermatomyositis shows pronounced IFN-I activity in muscle and skin, including MHC-I upregulation on myofibers; early clinical experience with JAK inhibition and pDC-targeted agents is encouraging [88]. In multiple sclerosis, exogenous IFN-β provides durable disease-modifying benefits within the CNS, whereas IFN-γ is associated with relapse biology, highlighting tissue-dependent directionality [89]. Type 1 diabetes displays an islet antiviral IFN-β program that may transition to pathogenic amplification under chronic stress, with CXCL10 and HLA class I hyperexpression as hallmarks [90]. Psoriasis involves an early pDC/type I IFN burst in lesions that crosstalks with the IL-23/Th17 axis; selective TYK2 inhibition is now approved. [91] In inflammatory bowel disease, IFN-λ generally confers epithelial antiviral protection but can impair mucosal repair during active inflammation; thus, agonism may be context-dependent [92]. Finally, genetic type I interferonopathies such as SAVI and AGS establish clear causality via constitutive pathway activation and respond to JAK inhibition, with next-generation cGAS–STING inhibitors emerging [93].

**Table 1:** Autoimmune diseases and evidence modalities for interferon involvement. Abbreviations: CLE, cutaneous lupus erythematosus; DM, dermatomyositis; IBD, inflammatory bowel disease; MS, multiple sclerosis; pSS, primary Sjögren’s syndrome; RA, rheumatoid arthritis; SLE, systemic lupus erythematosus; SSc, systemic sclerosis; SAVI, STING-associated vasculopathy; and T1D, type 1 diabetes. Modalities: H-Gen (human genetics), H-Obs (human observational/biomarker), H-Int (human interventional), M (mouse), and O (other ex vivo/in vitro).

Disease	Predominant IFN Axis	Key Source Cells	Confirmed Modalities (H-Gen/H-Obs/H-Int/M/O)	Hallmark Biomarkers	Therapeutic Notes
SLE/CLE [84,85]	Type I (α/β); keratinocyte IFN-κ in skin	pDCs, monocytes; keratinocytes (skin)	H-Obs, H-Int (IFNAR blockade), M, O	ISG score, SIGLEC1, CXCL10; cutaneous IFN-κ	Anifrolumab approved; pDC-targeting in late phase; TLR7/9 and IRAK4 inhibitors in development
Primary Sjögren’s syndrome (pSS) [86]	Type I » Type II (context)	pDCs, salivary epithelial cells	H-Obs, M, O	ISG signature in glands/blood; SIGLEC1; CXCL13/CXCL10	Investigational IFN pathway blockade; nucleic acid sensing targets under study
Rheumatoid arthritis (RA) [85]	Type II (γ) with Type I in subsets	T/NK cells (IFN-γ), synovial myeloid cells	H-Obs, M, O	GAS-driven transcripts; IFN-high synovial endotype	JAK inhibitors mitigate IFN-γ signaling; mixed data for direct IFN-I blockade
Systemic sclerosis (SSc) [87]	Type I dominant	pDCs, fibroblasts	H-Obs, M, O	Blood/skin ISG high; CXCL10; SIGLEC1	pDC and TLR inhibition being explored; antifibrotic combinations of interest
Dermatomyositis (DM) [88]	Type I dominant	pDCs, muscle/skin stromal cells	H-Obs, M, O	MHC-I upregulation; ISG in muscle/skin; myositis-specific Abs	JAK inhibitors used off-label; pDC/IFN-targeted trials ongoing
Multiple sclerosis (MS) [89]	Therapeutic Type I (β); Type II in relapse	CNS-resident cells, myeloid cells; T/NK (IFN-γ)	H-Int (IFN-β), H-Obs, M	Response to IFN-β; CSF chemokines	IFN-β approved; JAK/TYK2 under evaluation for subsets
Type 1 diabetes (T1D) [90]	Type I/II (islet antiviral tone vs. inflammation)	β-cells (IFN-β), myeloid and T/NK cells	H-Obs, M, O	Islet ISG; CXCL10; HLA class I hyperexpression	Early trials of JAK/TYK2; careful balance to preserve antiviral defense
Psoriasis [91]	Type I (lesional) with IL-23/Th17 axis	Keratinocytes, myeloid cells	H-Obs, M, O	Cutaneous ISG; IFN-κ; pDC-derived IFN early in lesions	TYK2 (deucravacitinib) approved; anti-IFN not standard
IBD (Crohn’s/UC) [92]	Type III (λ) at epithelium; Type I in subsets	Epithelial cells, myeloid cells	H-Obs, M, O	Epithelial ISG; mixed IFN-λ effects on repair	Caution with IFN agonism; pathway inhibitors investigational
Interferonopathies (e.g., SAVI, AGS) [93]	Constitutive Type I	Intrinsic (genetic activation in immune/stromal cells)	H-Gen, H-Int (JAK), H-Obs, M	Very high ISG; genetic diagnosis	JAK inhibitors clinically beneficial; cGAS–STING inhibitors emerging

### 3.1. IFNs in Autoimmune Rheumatic Diseases

ADs are the result of IR dysfunction leading to autoantibody synthesis, formation of immune complexes or CMI activation towards self-cells and inflammatory reactions mediated by neutrophils and monocytes, which are stimulators of IFN synthesis, especially IFN-I. Occasionally, the level of proinflammatory cytokines reaches high levels, leading to a complex pathology referred to as cytokine release syndrome (CRS). In some systemic ADs, IFNs are major components of the cytokine picture, influencing the course of the pathological process.

Measuring serum levels of IFNs poses major technical difficulties. Evaluating the expression of ISGs (IFN signature—IFN fingerprint) in circulating mononuclear leukocytes and target tissues of ADs and control group patients has low sensitivity because IFN-stimulated genes can be stimulated by other factors [94,95] and by other types of IFNs [96]. ELISAs gave uncertain and conflicting results about the level of IFN-α in serum, probably due to the interferences caused by the presence of other types of IFNs. Wilson et al. (2016) used the digital SIMOA (single-molecule array) method of ELISA technology, which detects IFN femtogram concentrations in human serum samples [97].

Molecular approaches can also assess the level of IRF-regulated and IFN-encoding gene transcription in PBMC: qRT-PCR and microarray allow simultaneous analysis of thousands of mRNA copies in a sample. The microarray method for the detection of IFN-activated genes (IFNGS) is sensitive and allows the selection of the ISG with the highest expression to calculate the “IFN score” as the average expression level compared to control samples [98], but has a drawback in that the PBMC population has individual numerical differences, the proportion of different cell types is different, and ISGs can be activated by different types of IFNs [99,100].

IFN-I, diverse molecules with pleiotropic effects on the IS, are bridges of innate and adaptive IS. Their primary role is antiviral protection. They have antiproliferative, including antitumor activity, in addition to a broad spectrum of functions, but also influence the pathological course of some ADs [101].

Dysregulation of IFN-I synthesis can lead to various pathologies, grouped as interferonopathies, triggered by self-nucleic acids, as a result of the IS’s inability to distinguish between self and non-self. Interferonopathies are closely associated with autoinflammatory disease [102]. Increased IFN-I levels have been detected in the serum of several ADs—SLE, RA, myositis, dermatomyositis, SS, autoimmune pancreatitis, systemic vasculitis, SS, and D1 [103,104]—while high expression of IFN-I-encoding and IFN-I-responsive genes (ISGs) was also observed [105,106]. The cytopathology of these patients is characterized by increased numbers of pDC, the histological marker of dysregulated IFN-I synthesis.

Clinically, all these ADs manifest with SLE-like symptoms and molecular features [107,108,109]. Prolonged synthesis of IFN-I over time or long-lasting activation of ISGs in circulating leukocytes and tissues in patients with ADs amplifies pathogenesis and clinical manifestations [101].

Increased serum levels of IFNs in some ADs are explained by mutations at all three levels of the gene chain that trigger and control the level of IFN synthesis: (1) mutations in cytosolic sensors for RNA or DNA, which cause chronic activation of the ISG activating signal transduction pathway; (2) mutations in ISGs that control the optimal level of IFNAR signaling [60]; and (3) mutations of IRFs.

The transcription of ISGs is amplified in response to the duration and intensity of the IFN signaling, as well as various intra- and extracellular factors [110]. The most important ISGs are those encoding leukocyte-attracting chemokines. A subset of ISGs is expressed only in response to IFN stimulation, whereas others are downregulated [111].

Chronic stimulation of IFN-I synthesis by mutations at any of the three steps appears to induce immune dysfunction: activating B cells and Ab synthesis, chemokine release and proinflammatory IL release in the mutually activating IFN-I-autoantibody-IL interaction [112,113,114].

Frequently, increased levels of IFN-I have contrasting effects—immunostimulatory or immunosuppressive—that manifest beneficially or detrimentally in the progression of ADs, depending on the activation pathway and the duration of transient or sustained ISG functionality [101,115].

The diversity of clinical manifestations of patients with systemic ADs and increased IFN-I levels is explained by the large number of IFN-activated genes (ISGs), estimated at 10% of the genome. Moreover, patients with the same ADs have different serum and tissue levels of IFN-I. Genes associated with increased risk of developing AI are polymorphic and account for an unknown proportion of total ISGs, while their transcription rate is modulated by signals transduced by IFN receptors [99]. On the other hand, genes potentially responsive to IFN signals encode cytokines that, by autocrine effect, modulate their own function [51].

Other factors that explain the diversity of IFN effects in ADs: IFNs induce epigenomic signatures through structural changes in chromatin and gene expression [116]; they activate latent promoters and inactivate others [117].

It would be possible that IFN-I is involved in early loss of tolerance, and the heterogeneous clinical manifestations could be attributed to tissue specificity [99]. ADs are categorized by dominant clinical manifestations, because the molecular triggering mechanisms are unknown. Rheumatic diseases with an immuno-inflammatory component—SLE, SS, DM, SSc—have in common a dysregulation of IFN synthesis, resulting in increased serum levels. Some ADs (SLE, SS) show a clear sexual bias. The clear preponderance of SLE pathogenic factor expression in a female–male ratio of 9:1 is attributed to sex hormones, which modulate immune cell functions.

Sjögren’s syndrome (SS) predominantly affects the female sex, with the ratio of female to male patients being 9:1. The sexual dimorphism in the incidence of some ADs is explained by the fact that female sex hormones stimulate genes that control innate immune reactivity. Patients with an extra X chromosome (Kleinfelter-XXY syndrome and trisomy X) have an increased risk of developing rheumatic AMD [118].

### 3.2. IFN-I in Systemic Lupus Erythematosus

The name SLE derives from the characteristic wolf bite-like facial lesions. It is a multisystemic syndrome with heterogeneous clinical manifestations, with a markedly higher incidence in women, especially during pregnancy (female to male ratio = 9:1), which argues the role of hormones in the activation of predisposing genes.

The etiopathogenesis of SLE is uncertain, due to the interaction of numerous genetic, epigenetic, hormonal, and environmental factors. Increased rates of lymphocyte apoptosis and macrophage deficiencies in phagocytosing apoptotic cells, as well as aberrant polarization leading to chromatin persistence in plasma, appear to be precursor conditions [119,120].

Genetic predisposition and the activation of cytosolic and endosomal sensors as a result of persistent self-DNA, due to deficiency in chromatin clearance released from lysed cells, are inducers of excessive synthesis of IFN-I.

SLE is an AD in which all three types of IFNs are synthesized, of which IFN-I has the highest level. A proportion of 50–70% of adult SLE patients express a marked increase in IFN-I. The increased serum level of IFN-I has an inherited genetic determinism and appears to be a risk factor for SLE, but also for other rheumatic ADs [118,121]. ISG activation has an undeniable role in disease progression.

The serum level of IFN-I correlates with active pathologic episodes (flare-ups) and consequently decreases during periods of remission [122]. SLE predisposing ISG alleles are transcribed at a higher rate in patients during pathology activation [123]. IFN-I activates mDCs displaying Ag of autoreactive lymphocyte clones and disrupts the peripheral tolerance state. The dominant immune dysfunction of SLE pathology consists of B cell activation, synthesis of specific autoantibodies against a wide diversity of Ag released from lysed cells, formation and persistence of immune complexes (ICs), their deposition in tissues and consequent organ dysfunction. Certain autoantigens are encoded by ISGs, whereas others include PRRs whose expression is induced by IFN signaling [124,125], suggesting IFN involvement in SLE pathogenesis. The effectors of pathogenesis are immune complexes consisting of Ab, DNA, RNA, and proteins, that are persistent in plasma. TLRs bind cytosolic DNA and induce IFN-α synthesis in pDCs. Neutrophil influx leads to NETosis, a form of programmed cell death. DNA in NETs contains hypomethylated sequences similar to bacterial and viral DNA, it is consequently endocytosed by macrophages and tissue pDCs and activates the intracellular NLRP3 sensor, inducing synthesis in IFNs I and II [126,127,128].

In the preclinical phase of SLE, pDCs are not effectors. The probably significant contribution to IFN-I production in the preclinical phase is attributed to keratinocytes through IFN-κ synthesis [108].

IFN-α stimulates DC maturation and differentiation of unengaged T cells into CD8^+^ and Th17 cells, while inducing Treg suppression. IFN-α-stimulated monocytes release BAF (B lymphocyte-activating factor).

Analysis of SLE pathology has been the subject of a large number of studies, which have contributed to the understanding and substantiation of the role of other types of IFNs in the pathology of different ADs.

**Figure 3:**
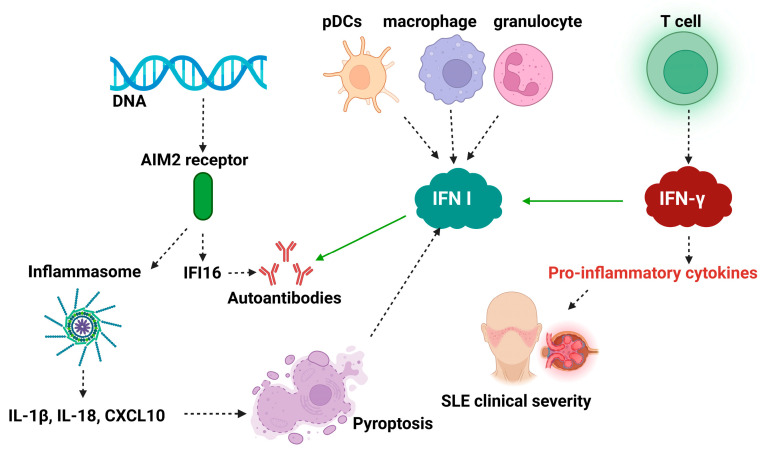
Pathogenic pathways driving the clinical phase of systemic lupus erythematosus (SLE). Cytosolic DNA is recognized by the AIM2 receptor, which activates two parallel pathways: (i) induction of IFI16, an autoantigen that promotes autoantibody production, and (ii) assembly of the inflammasome, leading to secretion of IL-1β, IL-18, and CXCL10, cytokines that contribute to disease severity. Plasmacytoid dendritic cells (pDCs), macrophages, and immature granulocytes are major sources of type I interferon (IFN-I), whose sustained production is a hallmark of SLE vascular pathology. IFN-I levels are further amplified by pyroptotic cell death, which releases danger signals. Effector T cells secrete IFN-γ, a stimulatory cytokine that induces additional proinflammatory mediators and precedes the rise in IFN-I and anti-DNA autoantibodies in the preclinical phase. Together, these interconnected pathways drive autoimmunity, cytokine dysregulation, and clinical manifestations of SLE, including skin and renal involvement.

The clinical phase of SLE results from the convergent effect of several factors: (1) IFN-I, a critical factor of vascular pathology in SLE, is found in high levels, being synthesized by pDCs, macrophages, and immature granulocytes [106]. (2) High levels and sustained increased expression of IFN-I synthesis activator pathway genes. (3) Proinflammatory cytokines released by effector cells of the autoantigen response reaction are immunosuppressive. For example, the ALR sensor (AIM 2) recognizes cytosolic DNA and induces the synthesis of IFN-I activator protein 16 (IFI 16). IFI 16 is an autoantigen that induces autoantibody synthesis. On the other hand, ALR, after binding a CARD (caspase activation and recruitment domain) containing protein, functions as an inflammasome, releasing IL-1β, IL-18, and CXCL10 (anti-inflammatory IL-10 binding chemokine), with immunosuppressive effect. Their serum level correlates with SLE severity [56]. (4) Cytokines induce cell death by pyroptosis, stimulating IFN-I synthesis [124,127,129]. (5) IFN-γ synthesized by effector cells of autoimmune reactivity is a stimulatory factor for the synthesis of proinflammatory cytokines. The synthesis of IFN-γ precedes both IFN-I synthesis and the increase in anti-DNA autoantibody titer [130], with all three components being present in the plasma of patients in the preclinical phase [131] (Figure 3).

SLE patients grouped according to specific clinical manifestations—mucocutaneous, cardiovascular—express the synthesis of a specific type of IFN-α, -γ or -λ [110]. Those with high levels of IFN-I have more complex pathologies, especially those with nephritis and arthritis with mucocutaneous and cardiovascular involvement [107,132].

### 3.3. IFN-I in RA

RA is a systemic, chronic inflammatory joint disease, and its manifestations predominantly involve the upper and lower limbs. Although RA is considered an autoimmune disease, characterized by the activation of TCD4 cells, which control the synthesis of autoantibodies, IFN-γ, and the inflammatory process, the pathological picture is clearly dominated by a severe and chronic inflammatory reaction of articular cartilage, stimulated by infectious Ag or superantigens, which may evolve to bone erosion. TCD4 cells and IFN-γ do not respond to mAbs, and serum rheumatoid factor does not precipitate with native IgG and does not meet the minimal criteria for self-reactivity [133].

In RA pathology, an important role is attributed to Th17 lymphocytes: they synthesize IL-17, stimulate the synthesis of chemokines and chemokine receptors in the synovial membrane, recruit lymphocytes and neutrophils, stimulate bone resorption, angiogenesis, and tissue destruction by stimulating VEGF and metalloproteases [134]. Neutrophils in the synovial fluid secrete proinflammatory cytokines—TNF, BAF (B cells activating factor)— and produce ROI, carry out cytotoxic effects, release lysosomal proteases, and show increased levels of MPO which they release into the blood and synovial membrane. MPO increases vascular permeability and thus proinflammatory cells penetrate tissues and generate NETs. NETs are made up of histones, DNA strands and citrullinated proteins, with the latter reportedly triggering AI processes [135].

In RA, experimental evidence suggests a bidirectional, pro- and anti-inflammatory, protective regulatory effect of IFN-γ [136,137].

### 3.4. IFN in Sjögren’s Syndrome

Sjögren’s syndrome (SS) is a chronic epithelial autoimmune disease, an exocrine disorder associated with a higher risk of developing NHL lymphoma [138]. The pathology of SS consists of the activation of lymphocytes and lysis of the epithelia of the salivary glands, lacrimal glands, and Meibomian glands (sebaceous glands of the lower eyelid margin that maintain the moisture of the cornea), resulting in a dry sensation of the cornea and oral mucosa. The serologic and histopathologic features of SS are increased salivary gland volume, decreased C4, germinal center formation, and macrophage infiltration of the minor salivary glands [138].

Serum IFN-I, as well as the expression of the genes it activates, is elevated in salivary glands [139], but is not attributed to a pathogenic role. In biopsies from lesions of minor salivary glands, microarrays revealed increased activity of ISGs. A significant role in SS pathology is attributed to IFN-γ. The mRNA of IFN-γ in biopsies of minor salivary glands was significantly increased in SS-lymphoma NHL patients [98,140].

In macrophages and serum, IHC revealed the presence of IFN-λ. The mechanisms of activation of IFN-λ synthesis are common with IFN-I, suggesting the proinflammatory effect of IFN-λ [141].

### 3.5. IFN in Systemic Sclerosis

Systemic sclerosis (SSc-scleroderma), an AD of unknown etiology, is characterized by dysregulation of immune function, innate IS activation, vasculopathy, and fibrosis of the tegument and viscera. It produces the highest mortality (3.5%) of all rheumatic diseases due to excessive, multifactorial fibrotic response.

Chronic stress-induced tissue injury of tissue fibroblasts is the triggering factor of SSc, in which myeloid cells—monocytes, macrophages, and mDCs—stimulate collagen synthesis and appear to play a key role in fibrotic pathology that alters tissue architecture in the tegument, kidneys, heart, lungs and GI tract. Dendritic cells (DCs), which originate in the bone marrow, play a key role in presenting antigens released by injured fibroblasts and in producing cytokines that stimulate fibrogenesis, particularly in the context of defective cellular repair mechanisms [59,142]. Fibrogenesis induces synthesis of IFN-I. The two function in a mutually stimulatory interaction [143,144,145]. Transcription of IFN-I-encoding genes is regulated to IRFs 1-9, their stimulatory potential being heterogeneous. In SSc and SLE alike, activation of IRFs 4, -5, -7, -8 [146] is considered a risk factor and similar to STAT4, generates increased levels of IFN-I in the plasma of SSc and SLE patients [143,144,147].

Increased plasma levels of IFN-I were seen in 50% of SSc and SLE patients. Different cell types of the affected organs express different levels of IFN-I-activated gene expression [148]. Circulating pDCs, reduced in number, infiltrate the skin and synthesize chemokines that recruit neutrophils and monocytes. Monocytes differentiate into pDCs and macrophages, which synthesize IFN-I and IL-2, with proinflammatory action. IFN-I activates the synthesis of IL-6, the cytokine that initiates fibrosis [143,148]. Another IFN-I stimulatory factor is DNA released from mitochondria injured by oxidative stress. Mitochondrial DNA increases in the plasma of SSc and SLE patients, but also reaches endosomes and stimulates IFN-I synthesis [115]. In the serum of SSc patients, as in COVID-19 patients with persistent inflammation, IFN-λ concentration increases.

In total, 50% of SSc patients have elevated serum IFN-I levels from the early phase of the disease. Increased IFN-λ is detectable later but does not appear to have a pathological effect.

Polymyositis and dermatomyositis are disorders of dense connective tissues such as tendons and ligaments. Increased levels of IFN-α have been observed in patient sera, with ISGs, such as MX1, detected in muscle and skin tissues, alongside infiltrating pDCs. MX 1 encodes a protein with an inhibitory role in the replication of some RNA and DNA viruses [149].

### 3.6. IFN-λ in ADs

Under physiological conditions, IFN-λ has a protective antiviral role by stimulating expression of MHC complex molecules in mDC, expression of PRRs on the pDCs, and Ab synthesis by plasmocytes, while suppressing Treg activity. Autoimmune reactivity is characterized by the same pattern of immune stimulation, reflected in the increased serum levels of IFN-I and IFN-II in SLE, RA, CBI, SSc, and SS patients [150].

IFN-λ stimulates IFN-γ synthesis and amplifies the chronic inflammatory process. pDCs, which present elevated numbers in rheumatic diseases, have receptors for IFN-λ with autocrine action. In patients with SLE, IFN-λ activates keratinocytes and mesenchymal cells, which release chemokines that recruit neutrophils, bind to their receptors, stimulate inflammation and may be considered stimulatory factors in AI pathogenesis [113,151].

In RA, IFN-λ is proinflammatory: it stimulates the synthesis of IL-6, IL-8, and metalloproteases. IFN-λ inhibits the differentiation of Th2 lymphocytes and stimulates Th1 polarization, with an indirect proinflammatory effect.

In the sera of RA patients, that is, in those with ankylosing spondylitis, IFN-λ is elevated and also detectable in synovial fluid [113].

### 3.7. IFNs in Other ADs

Autoimmune pancreatitis, a chronic destructive inflammatory condition probably associated with systemic ADs, is considered an AI issue due to the lymphocytic infiltrate. Its pathology is characterized by inflammation and a fibrosing response induced by IFN-I and IL-33, whose synthesis is induced by IFN-I [105,152].

Psoriasis (=pruritus) and atopic dermatitis are the most common autoimmune skin diseases mediated by T cells. The pathogenesis of psoriasis appears to be triggered by tegument trauma (wounds, burns, surgical incisions, tattoos). Cytokines released in traumatic injury stimulate an ample influx of pCDs, which release IFN-α, the main trigger, while IFN-β produced by keratinocytes stimulates pDC activation and maturation [153,154].

Clinically, psoriasis is a chronic inflammatory condition. Histologically, it is characterized by hyperproliferation of keratinocytes, dysfunction of the epithelial barrier associated with dermal infiltration of activated T cells, and production of proinflammatory cytokines [135]. Excess keratinocytes lead to erythematous dermal lesions, which evolve into scaly, silvery structures. Plaque-like lesions may be localized or cover extensive tegumental areas, caused by breakdown of the stratum corneum.

Phenotypically similar skin lesions in psoriasis can arise via two distinct mechanisms. The classic form is mediated by T lymphocytes under TNF control. In contrast, “psoriasis-like” lesions emerge during treatment with anti-TNF antibodies, which is the standard therapy for rheumatoid arthritis, Crohn’s disease, and psoriasis. In these cases, TNF is absent, and inflammation is driven instead by type I interferons (IFN I) produced by plasmacytoid dendritic cells (pDCs), likely activated by microbial antigens [155].

In patients with psoriasis, TGF β promotes Th17 cells. Immunofluorescence studies on skin biopsies show co-localization of RORγt^+^ Th17 cells with IFN λ. These Th17 cells produce IL 29, an antiviral cytokine. In psoriatic lesions, IFN λ–induced ISG activation and elevated levels of chemokines that attract NK cells, CD8^+^ T cells, and CD4^+^ T cells were observed. Further research by Wang et al. (2021) revealed that psoriatic lesions also contain IFN I mRNA—produced by infiltrating pDCs—as well as the receptors IFNAR1 and IFNAR2, completing the picture of psoriasis immunopathogenesis [153,156].

Inflammatory bowel diseases (IBDs), including Crohn’s disease and ulcerative colitis (UC), are chronic, relapsing inflammatory disorders of the intestinal tract. Histologically, both conditions exhibit ulcerative lesions driven by persistent inflammation characterized by dense infiltration of macrophages, lymphocytes, and numerous plasma cells within the intestinal wall.

Ulcerative colitis is a chronic idiopathic condition confined to the mucosal layer of the colon, where inflammation is typically continuous. In contrast, Crohn’s disease is a transmural, segmental pathology that can affect any part of the gastrointestinal tract, though it most commonly involves the colon and small intestine. In Crohn’s, the inflammatory infiltrate may extend beyond the mucosa into the submucosa and muscularis and often forms granulomas. The disease pattern is patchy, with areas of healthy tissue alternating with inflamed segments.

Both UC and Crohn’s disease are associated with increased intestinal permeability, facilitating translocation of microbial products such as lipopolysaccharide (LPS) and bacterial DNA, which fuel chronic immune activation. Patients exhibit elevated plasma levels of proinflammatory cytokines and immunoglobulins (IgG, IgM, IgA). Notably, in UC, IgG1 and IgG3—potent activators of the complement system—are prominently secreted. Their activity leads to the release of complement components C3a and C5a, which recruit inflammatory cells to deeper mucosal layers [157].

Within the intestinal lamina propria, CD4^+^ T cells dominate and secrete high levels of proinflammatory Th1 cytokines such as IL-12, IFN-γ, and TNF, further amplifying the inflammatory response and contributing to tissue damage.

Intestinal epithelial cells, which form the primary interface between the gut microbiota and the immune system, play a pivotal role in the pathogenesis of IBD. Epithelial dysfunction disrupts the delicate balance between microbial antigens and the immune response, altering the interactions between the microbiota and innate immune cells.

However, the role of IFN λ in IBD remains unclear, largely due to the contrasting effects of its subtypes in viral infections [158]. These divergent outcomes are influenced by regulatory factors controlling IFN synthesis and by activators of ISGs. Among these, IRFs play a central role in modulating innate immunity, acting either cooperatively or competitively. The functional diversity among the nine IRF family members (IRF1–9) helps explain the varied and sometimes opposing effects of IFN λ signaling in intestinal inflammation [159].

IFN-γ, initially regarded as the prototypical proinflammatory cytokine, is now recognized as a pleiotropic immunomodulator capable of exerting both pro- and anti-inflammatory effects, depending on the tissue-specific microenvironment. IFN-γ enhances the metabolic activity of macrophages and neutrophils, promoting the release of ROIs and augments innate immune responses. It also upregulates MHC class I and II expression, facilitates adaptive immunity, and drives CD4^+^ T cell differentiation into the Th1 phenotype. In addition, IFN-γ inhibits cell proliferation, promotes apoptotic cell death, and enhances IL-12 expression, thereby amplifying its own production through a positive feedback loop.

The dual role of IFN-γ has been documented in central nervous system (CNS) pathology, particularly in multiple sclerosis (MS) and its animal model, experimental autoimmune encephalomyelitis (EAE); both are chronic inflammatory disorders characterized by immune infiltration, demyelination, and neuronal damage [160,161]. In these models, IFN-γ exerts neuroprotective or neurotoxic effects depending on its cellular source and the disease stage.

In Sjögren’s syndrome (SS), IFN-γ production in minor salivary gland biopsies is temporally coordinated with type I interferon (IFN-I) synthesis [96]. As the disease progresses, shifts in the proinflammatory cell populations and cytokine expression ratios help explain the seemingly contradictory roles of IFN-γ.

In EAE, for example, IFN-γ has been shown to enhance Foxp3 expression in regulatory T cells (Tregs), promoting immunosuppression and limiting disease severity. Conversely, IFN-γ produced by γδ T cells exacerbates demyelination, illustrating the context-dependent nature of its effects [161].

Multiple sclerosis (MS) is the most common non-traumatic, disabling autoimmune disorder of the central nervous system (CNS) in young adults. Although there is a genetic predisposition, the onset and progression of MS are strongly influenced by environmental factors, including vitamin D deficiency, smoking, childhood obesity, and most notably, Epstein–Barr virus (EBV) infection [162]. MS is initiated by an autoimmune response, which is subsequently amplified by a chronic inflammatory process targeting the brain and spinal cord. Pathologically, MS is characterized by perivenular inflammation, predominantly driven by CD8^+^ T lymphocyte infiltration, with a lesser but significant contribution from B lymphocytes and plasma cells. Infiltrating B cells contribute to CNS inflammation through the production of proinflammatory cytokines, particularly interleukin-6 (IL-6) [35].

Oligodendrocyte-derived myelin sheaths are destroyed by microglia, the resident macrophages of the CNS. These demyelinated lesions, or plaques, are histologically identified by the accumulation of myelin debris within macrophages. Over time, demyelinated axons fail to remyelinate, leading to irreversible sensory and motor deficits, typically manifesting in adults aged 20–40 years.

In vitro, lymphocytes from MS patients release increased amounts of IFN-γ. In vivo, IFN-γ acts as a cofactor of demyelination by increasing MHC expression on microglia, amplifying anti-myelin IR and the pathological process overall [163].

Type 1 diabetes (T1D) is an AD that primarily affects children and young adults, though approximately 32% of cases occur after the age of 30. The autoimmune response leading to T1D is triggered by a combination of intrinsic (genetic) and extrinsic (environmental) factors. While genetic predisposition plays a central role, environmental triggers, most notably Coxsackie B virus (CVB) infection, along with epigenetic modifications, MHC class I overexpression, and endoplasmic reticulum (ER) stress, contribute to the initiation of autoimmune β-cell destruction [164].

The MHC located on chromosome 6p21 contains over 200 alleles involved in antigen presentation via MHC classes I, II, and III. MHC class I molecules present antigens to CD8^+^ T cells, while MHC class II molecules interact with CD4^+^ T cells. Genetic susceptibility to T1D is strongly associated with the presence of HLA-DR3-DQ2 and/or DR4-DQ8 alleles, with the highest risk observed in individuals who are DR3/DR4 heterozygotes [90,165].

A key player in the detection of viral infection in pancreatic islet cells is protein kinase R (PKR), a cytoplasmic kinase activated by double-stranded RNA (dsRNA). PKR expression is induced by IFN-I, particularly IFN-α, which plays a central role in the early antiviral response (Gal-Ben-Ari, 2018 [166]). PKR is one of the four mammalian kinases that serve as dsRNA sensors, initiating downstream signaling that can lead to β-cell apoptosis [167].

Coxsackie B virus (CVB) infection induces the production of IFN-α as part of the essential antiviral immune response. However, in individuals with a genetic predisposition to T1D, IFN-α appears to act as a critical trigger of the autoimmune response. Specifically, IFN-α (1) upregulates MHC class I expression on pancreatic β-cells, enhancing their visibility to CD8^+^ T cells; (2) induces the production of chemokines that attract cytotoxic T lymphocytes (CTLs) and natural killer (NK) cells to the islets; (3) stimulates autoimmune reactivity against β-cells; (4) promotes endoplasmic reticulum (ER) stress; and (5) inhibits insulin synthesis, further contributing to β-cell dysfunction and destruction [168].

Evidence for type I IFN activation in T1D pathogenesis is supported by the expression of MxA, PKR, and HLA class I molecules in islets positive for anti-islet autoantibodies, and especially in CVB-infected islets [164,169].

The proinflammatory role of interferon-gamma (IFN-γ) is well established in autoimmune diseases and conditions characterized by intense immune activation. In type 1 diabetes (T1D), the destruction of the islets of Langerhans is primarily mediated by CD8^+^ cytotoxic T lymphocytes, whose cytolytic activity is enhanced by Th1 lymphocytes, a process strongly associated with elevated IFN-γ expression [170].

The apoptotic loss of insulin-producing β-cells is most pronounced during the preclinical phase of T1D, in which IFN-γ plays a central pathogenic role. Together with IL-1β and TNF-α, IFN-γ contributes to β-cell apoptosis and functional impairment. Mechanistically, IFN-γ (1) upregulates MHC class I expression on β-cell surfaces, enhancing their recognition by CD8^+^ T cells; (2) induces chemokine receptor expression on CD8^+^ T cells, promoting their targeted migration (“homing”) to pancreatic islets; and (3) amplifies the autoimmune response against β-cells [171].

This synergistic effect of IFN-γ and TNF-α in promoting immune-mediated tissue damage has also been observed in SARS-CoV-2 infection, highlighting a broader mechanism of inflammatory synergy [172].

## 4. Clinical Translation and Therapeutic Landscape

### 4.1. From Association to Causation in IFN Biology

Many datasets have linked elevated IFN levels or ISG “signatures” with autoimmune phenotypes, but it is important to emphasize that correlation does not necessarily imply causation. Association studies remain valuable for identifying potential biomarkers and endotypes, yet only a smaller number of investigations provide evidence for direct mechanistic involvement of interferon pathways. Stronger causal inference can be drawn when several criteria are met. Longitudinal cohort studies in systemic lupus erythematosus (SLE) have shown that surges in type I IFN and ISG expression precede disease flares, suggesting a triggering rather than a secondary role in pathology [173]. Human genetics also offers natural experiments: monogenic interferonopathies such as TREX1-, ADAR1-, or STING1-associated disorders are caused by constitutive IFN-I signaling and result in inflammatory phenotypes, while therapeutic improvement with JAK inhibition in syndromes such as SAVI underscores the pathogenic role of this pathway [174]. In addition, gain-of-function mutations in TLR7 have been shown to directly cause human SLE, thereby firmly linking nucleic acid sensing, IFN activation, and autoimmunity [175]. Perhaps the most convincing causal evidence comes from interventional studies. Inhibition of IFN signaling with the anti-IFNAR1 antibody anifrolumab has produced clinical improvement in SLE and received regulatory approval, demonstrating that IFN activity drives pathology in at least a subset of patients [176]. Moreover, pharmacodynamic analyses indicate that patients with an IFN-high endotype respond better to such treatments, further supporting a dose–response and specificity relationship [177]. Collectively, these findings justify a framework in which IFN signatures are reported as either association, strong causal inference, or proven causality, depending on the nature of the evidence.

In parallel with the mechanistic insights, clinical translation of IFN biology has advanced significantly. The most notable milestone has been the approval of anifrolumab for moderate-to-severe SLE, based on the Phase 3 TULIP program and earlier trials that demonstrated reductions in global disease activity, cutaneous manifestations, and corticosteroid use [178]. Earlier anti-IFN-α monoclonal antibodies such as sifalimumab and rontalizumab provided important proof-of-mechanism by lowering ISG levels, although they did not progress to licensure [179]. Upstream targeting of plasmacytoid dendritic cells, the principal producers of type I IFN, is also under investigation. The anti-BDCA2 antibody litifilimab (BIIB059) reduced IFN production and improved cutaneous lupus in Phase 2 trials, and late phase studies are ongoing [180]. Beyond direct IFN blockade, inhibition of downstream JAK–STAT signaling has been successfully applied in monogenic interferonopathies such as SAVI, where ruxolitinib and baricitinib ameliorated inflammatory and pulmonary manifestations, though these drugs have broader immunological effects beyond IFN signaling [181]. Selective TYK2 inhibitors, such as deucravacitinib, also act on proximal IFN pathways and have been approved in psoriasis, with potential relevance for autoimmune conditions characterized by interferon signatures [182].

Interestingly, interferon agonists illustrate the dual nature of this pathway in disease. Type I IFN therapy has long been a mainstay of multiple sclerosis treatment, where IFN-β exerts anti-inflammatory effects in the central nervous system [183]. Type III IFNs, such as IFN-λ, are being evaluated primarily as antivirals, for example, in hepatitis and COVID-19, but preclinical studies indicate that their effects in autoimmunity are complex, balancing protective antiviral responses against potential impairment of mucosal repair and promotion of epithelial pyroptosis [184].Taken together, the totality of evidence supports a causal role for interferon pathways in SLE and selected interferonopathies, especially in patients with high baseline IFN signatures, while also emphasizing the tissue-specific and sometimes protective roles of interferons in other contexts. Future translation will likely require biomarker-guided stratification to match patients with the appropriate IFN-targeted intervention [184]. These observations highlight the context-dependent consequences of interferon signaling.

Taken together, the totality of evidence supports a causal role for interferon pathways in SLE and selected interferonopathies, especially in patients with high baseline IFN signatures, while also emphasizing the tissue-specific and sometimes protective roles of interferons in other contexts. Future translation will likely require biomarker-guided stratification to match patients with the appropriate IFN-targeted intervention [185].

### 4.2. IFN Pathway Therapies Under Clinical Development or Approved

The therapeutic landscape targeting interferon pathways spans direct blockade of interferons, inhibition of their receptors, modulation of upstream producers such as plasmacytoid dendritic cells, and inhibition of downstream signaling molecules [186]. The first-in-class anti-IFNAR1 antibody anifrolumab has been approved for systemic lupus erythematosus, following the positive results of the Phase 3 TULIP-1 and TULIP-2 trials and the Phase 2 MUSE study, which demonstrated improvements in global disease activity, skin and joint involvement, and corticosteroid tapering [187] (Table 2). Other monoclonal antibodies targeting IFN-α, such as sifalimumab and rontalizumab, showed suppression of ISG signatures and early clinical signals in SLE but were discontinued before late phase development [188,189].Upstream modulation of IFN production is represented by litifilimab (BIIB059), a monoclonal antibody against BDCA2 on plasmacytoid dendritic cells, which lowers IFN-I production and showed clinical benefits in cutaneous lupus erythematosus during the Phase 2 LILAC trial [190]; Phase 3 trials are ongoing in SLE and CLE.

Downstream signaling inhibition through JAK inhibitors (such as ruxolitinib, baricitinib, and tofacitinib) has been particularly successful in monogenic interferonopathies like STING-associated vasculopathy (SAVI), where they improved systemic and pulmonary manifestations [191]. Although these agents are not IFN-specific, their broad efficacy highlights the central role of JAK–STAT in mediating interferon effects. Selective TYK2 inhibitors such as deucravacitinib (recently approved in psoriasis) also block pathways downstream of type I IFN, IL-12, and IL-23, and represent an emerging class with relevance to interferon-driven disease [192].

Conversely, interferon agonists remain clinically valuable in selected contexts. IFN-β has long been an established disease-modifying therapy in multiple sclerosis, where it exerts anti-inflammatory and immunomodulatory effects. IFN-λ agonists are in clinical development mainly as antivirals, with promising results in hepatitis and COVID-19; however, their potential role in autoimmunity is still uncertain, as preclinical models suggest both protective and detrimental effects depending on the tissue and inflammatory context.

**Table 2:** Overview of Current and Emerging Interferon-Pathway Modulators in Autoimmune Disease.

Agent/Class	Target/Mechanism	Indication(s) Tested	Trial Phase/Status	Key Outcomes
Anifrolumab	Anti-IFNAR1 mAb (blocks type I IFN receptor)	SLE	Phase 3 → Approved	Reduced disease activity, skin/joint benefit, steroid sparing.
Sifalimumab/Rontalizumab	Anti-IFN-α mAbs (neutralize IFN-α subtypes)	SLE	Phase 2 (terminated)	ISG suppression, modest efficacy signals.
Litifilimab (BIIB059)	Anti-BDCA2 mAb (inhibits pDC IFN-I production)	CLE, SLE	Phase 2 → Phase 3	Reduced cutaneous disease activity, lowered IFN-I outputs.
JAK inhibitors (ruxolitinib, baricitinib, tofacitinib)	Block JAK1/2/3 signaling downstream of IFNAR	SAVI, interferonopathies, SLE (investigational)	Approved in other indications; proof-of-concept in IFN-driven disease	Improved systemic and pulmonary disease in interferonopathies.
Deucravacitinib	Selective TYK2 inhibitor (IL-12/23 and IFN signaling)	Psoriasis; investigational in other autoimmune diseases	Approved in psoriasis	Effective in TYK2-driven inflammation; potential relevance to IFN-driven autoimmunity.
IFN-β	IFN agonist	Multiple sclerosis	Approved	Longstanding disease-modifying therapy; anti-inflammatory effects.
IFN-λ agonists	Type III IFN agonists	Hepatitis, COVID-19	Phase 2–3 (not autoimmune)	Antiviral protection; uncertain role in autoimmunity.

Overall, these therapies highlight the dual nature of interferons: pathogenic drivers in some settings and protective mediators in others. Biomarker-driven patient stratification, particularly based on interferon gene signatures, will be crucial for optimizing clinical translation.

### 4.3. Future Directions

Despite major advances, several knowledge and translation gaps remain. First, we need standardized, cross-platform IFN assays that harmonize results across centers and sample types; the field would benefit from an external quality-assured short ISG panel coupled to an ultrasensitive IFN-α/β protein assay and a flow-based SIGLEC1 readout. Second, tissue resolution is paramount: integrating spatial transcriptomics with multiplex imaging can identify the cellular sources and targets of IFNs within inflamed organs, clarifying why the same pathway is pathogenic in one tissue yet protective in another. Third, endotype-matched trials should become the norm; enrichment by IFN score, pDC activation, or nucleic acid-sensing variants will increase study power and reveal responders versus non-responders. Fourth, we must delineate combination strategies—for example, pDC-targeting plus receptor blockade or IFN pathway inhibition layered with B cell modulation—using adaptive designs with biomarker-based futility rules. Fifth, next-gen therapeutics that spare antiviral competence while quelling autoimmunity (biased IFNAR antagonism, TBK1/IKKε tuning, tissue-tropic delivery) warrant platform trials with shared control arms and harmonized pharmacodynamic endpoints. Sixth, long-term safety requires registries that track infection phenotypes, vaccine responses, and latent virus reactivation under chronic IFN pathway inhibition. Finally, systems models linking circulating biomarkers to tissue circuitry—parameterized with longitudinal clinical data—could forecast flare risk and guide pre-emptive, time-limited dosing rather than continuous suppression.

Looking forward, progress in ADs will hinge on precision immunomodulation anchored in endotype-guided therapy rather than one-size-fits-all suppression. Clinically actionable biomarker frameworks should combine ultrasensitive protein interferons (digital IFN-α/β assays), compact IFN gene signatures, chemokines such as CXCL10 (IP-10), and cellular readouts including SIGLEC1 (CD169) and phospho-STAT signaling after standardized stimulation. Layering genomic risk (e.g., *TLR7*, *STAT4*, *IRF5*, *TYK2*, *TREX1*/*ADAR1*/*STING1*), autoantibody profiles, and single-cell/spatial multi-omics will refine patient stratification and reveal tissue-specific IFN circuits that dictate whether blocking, tuning, or leveraging interferons is most appropriate. Therapeutically, next-generation approaches should explore ligand- or receptor-biased IFN blockade, pDC-directed agents, inhibitors of nucleic acid sensing (TLR7/9, cGAS–STING, IRAK4), and selective signal-node modulators (e.g., TYK2, TBK1/IKKε), alongside context-specific IFN agonism where protective (e.g., IFN-β in CNS).

Clinical trial design must evolve accordingly. Biomarker-enriched, adaptive platform trials that pre-specify IFN-high inclusion, embed on-treatment pharmacodynamics (protein IFN, ISG suppression, pSTAT normalization) as dose-finding gatekeepers, and test mechanism-matched combinations (for example, pDC modulation plus IFNAR blockade, or IFN pathway inhibition layered with B cell-directed therapy) will accelerate go/no-go decisions and reduce exposure to ineffective regimens. Safety frameworks should proactively track infection phenotypes, vaccine responsiveness, and latent virus reactivation via integrated registries and real-world data streams. Finally, computational disease maps that fuse longitudinal clinical trajectories with multi-omic IFN metrics can enable forecasting of flare risk and support timed, tissue-tropic dosing strategies that deliver maximal efficacy with minimal loss of host defense. Together, these directions position interferon biology not merely as a correlate of autoimmunity but as a tractable axis for individualized intervention.

## 5. Conclusions

Autoimmune diseases have a genetic substrate, influenced by a yet-to-be-defined number of predisposing genes, and are primarily characterized by dysregulated immune reactivity and the release of proinflammatory cytokines. The pathology of immune-mediated autoimmune diseases is typically driven by the production of autoantibodies (auto-Abs) and the chronic inflammatory response accompanying autoimmune activation.

The nature of the initiating antigen(s) in autoimmune responses appears to influence the type of interferons (IFNs) produced. However, it remains unclear whether, in genetically predisposed individuals, IFNs act as primary triggers of autoimmunity, or whether their role is limited to amplifying and sustaining the inflammatory pathology, as seen in interstitial lung disease (ILD). In systemic lupus erythematosus (SLE), the prototypic rheumatic disease associated with ILD, elevated type I IFN levels preceding clinical onset strongly suggest a triggering role. In SLE, serum type I IFN levels and interferon-stimulated gene (ISG) expression correlate with disease severity, supporting the hypothesis that proinflammatory IFNs contribute to both the onset and propagation of ILD.

Building on the growing mechanistic understanding of interferon networks, future research should focus on biomarker-guided patient stratification to identify individuals most likely to benefit from interferon-targeted or interferon-modulating therapies. Multi-omics approaches—integrating transcriptomics, proteomics, and metabolomics—are expected to refine prediction of IFN responsiveness and treatment outcomes across autoimmune diseases. Furthermore, the development of next-generation therapeutics targeting nucleic acid sensors (e.g., TLR7, cGAS, STING) or key IFN-pathway nodes (e.g., JAK1, TYK2) holds promise for more selective and tissue-specific immunomodulation with reduced systemic toxicity. Collectively, these strategies represent essential steps toward precision immunotherapy in interferon-driven autoimmunity.

Interferons remain central orchestrators of immune homeostasis and autoimmunity, bridging antiviral defense and chronic inflammation. Despite substantial progress in mapping interferon signatures across autoimmune diseases, major gaps persist in distinguishing protective from pathogenic signaling pathways. Several key areas for future investigation exist: decoupling protective versus pathogenic IFN signaling, elucidating tissue-specific interferon effects, understanding persistence mechanisms that sustain chronic interferon activity, and promoting the integration of interferon biomarkers into precision immunotherapy frameworks. Advancing these priorities through multi-omics approaches and targeted therapeutic development will be pivotal for achieving precision modulation of the interferon system in human disease.

## Data Availability

No new data were created or analyzed in this study.

## Abbreviations

The following abbreviations are used in this manuscript:

AD	Autoimmune Disease
AIM2	Absent in Melanoma 2
ALR	AIM2-Like Receptor
ATRA	All-Trans Retinoic Acid
BBB	Blood–Brain Barrier
CD	Dendritic Cells
cDC/pDC	Conventional/Plasmacytoid Dendritic Cells (CDm(p)
UC	Ulcerative Colitis
DAMP	Damage-Associated Molecular Pattern
EAE	Experimental Autoimmune Encephalomyelitis
ER	Endoplasmic Reticulum
GM-CSF	Granulocyte–Macrophage Colony-Stimulating Factor
GI Tract	Gastrointestinal Tract
IFNGS/ISG	IFN Gene Signature/Interferon-Stimulated Genes
IL	Interleukins
CMI/RIMC	Cell-Mediated Immunity
IRF	Interferon Regulatory Factor
ISRE	Interferon-Stimulated Response Element
JAK1	Janus Kinase 1
lncRNA	Long Non-Coding RNA
miRNA	Micro RNA
MPO	Myeloperoxidase
MS	Multiple Sclerosis
NETs	Neutrophil Extracellular Traps
NF-κB	Nuclear Factor kappa-light-chain-enhancer of activated B cells
NLR	NOD-Like Receptors
NOD	Nucleotide-binding Oligomerization Domain
PAMP	Pathogen-Associated Molecular Pattern
PBMC	Peripheral Blood Mononuclear Cells
PCD	Programmed Cell Death
PRR	Pattern Recognition Receptors
RA	Rheumatoid Arthritis
IR/RI	Immune Response
RIP	Primary Immune Response
RLR	RIG-I-Like Receptors (Retinoic acid Inducible Gene-I-like Receptors)
ROI	Reactive Oxygen Intermediates
SI	Immune System
SLE	Systemic Lupus Erythematosus
SLO	Secondary Lymphoid Organs
SS	Sjögren’s Syndrome
SSc	Systemic Sclerosis
STAT	Signal Transducers and Activators of Transcription
STING	Stimulator of Interferon Genes
T1D	Type 1 Diabetes
TGF-β	Transforming Growth Factor Beta
TLR	Toll-Like Receptors
Treg	Regulatory T Cells
TYK2	Tyrosine Kinase 2
VEGF	Vascular Endothelial Growth Factor
